# Serum microRNA panel for early diagnosis of the onset of hepatocellular carcinoma

**DOI:** 10.1097/MD.0000000000005642

**Published:** 2017-01-13

**Authors:** Ying Zhang, Tao Li, Yumin Qiu, Tao Zhang, Pengbo Guo, Xiaomin Ma, Qing Wei, Lihui Han

**Affiliations:** a Department of Immunology, Shandong University School of Medicine; b Department of Infectious Diseases, Provincial Hospital Affiliated to Shandong University; c Department of Epidemiology and Biostatistics, School of Public Health, Shandong University, Jinan, China.

**Keywords:** early diagnosis, α-fetoprotein, hepatocellular carcinoma, serum microRNA

## Abstract

Unique change of circulating microRNAs (miRNAs) was recognized to occur in early oncogenesis, which conferred it the potential as biomarkers for early detection of cancer. However, its diagnostic capability for hepatocellular carcinoma (HCC) has not been fully understood. In this study, microarray analysis was applied to screen the initial candidate miRNA from both the supernatants of anoikis-resistant cellular models and the sera samples of HCC patients. The selected differentially expressed miRNAs were further verified in 115 HCC patients and 40 health controls by qRT-PCR. Among these, 4 miRNAs (miR-16-2-3p, 92a-3p, 107, and 3126-5p) were significantly changed in HCC patients compared with controls. Logistic regression analysis identified a 3-miRNA panel (miR-92-3p, miR-107, and miR-3126-5p) as valuable diagnostic marker for HCC, especially for early stage patients (AUC = 0.975) and for low-level AFP HCC patients (AUC = 0.971). In addition, the combination of 3-miRNA panel and AFP was even more effective for discriminating the early stage HCC patients (AUC = 0.988) and low-level AFP HCC patients (AUC = 0.989) from control. In conclusion, diagnostic efficacy of the combination of 3-miRNA panel and AFP was powerful for HCC diagnosis, especially in early tumor screening and low-level AFP patients.

## Abstract 2

Supplemental Digital Content is available in the text

## 1 Introduction

Hepatocellular carcinoma (HCC), one of the poorest prognostic solid tumors, is rated the 6th in incidence and 3rd in mortality worldwide.^[1]^ The poor prognosis of this disease is partially due to lack of effective means for early diagnosis. Consequently, curative treatments are no longer feasible because of intra- and extra-hepatic metastases at the time of diagnosis.^[2]^ Currently, the combination of serum alpha-fetoprotein (AFP) and ultrasounds surveillance is the most widely used strategy for screening and detection of HCC in high risk group.^[3]^ However, ultrasound surveillance is limited by sensitivity of the detection and often results in misdiagnosis of small malignant nodules, especially in cirrhosis. AFP is also an unsatisfactory marker for the detection of early onset of HCC due to the low sensitivity and high false-positive rate.^[4]^ Consequently, novel effective and reliable tools for detecting and diagnosing the early onset of hepatic carcinoma are highly desirable.

MicroRNAs (miRNAs) are endogenous small noncoding RNAs, which play important regulatory roles in various biological processes such as cell development, differentiation, and proliferation.^[5]^ It is reported that more than 50% of the miRNAs genes are located at cancer-associated genomic regions or fragile sites.^[6]^ The aberrant expression of miRNAs, which has been confirmed in various types of malignancies, contributes to oncogenesis and cancer progression.^[7]^ MiRNAs can circulate in a cell-free form in body fluids including serum and plasma, most probably in exosomes or protein-RNA-complexes, which protect them against degradation by RNase and other harsh conditions.^[8]^ Due to their inherent stability, ease of sampling by minimally invasive methods, and their proven role in tumorigenesis, circulating miRNAs hold great promise as novel noninvasive biomarkers for the diagnosis and assessment of HCC. However, the diagnostic value of circulating miRNAs for early diagnosis of HCC remained to be fully proven.

Acquisition of anoikis-resistance is the hallmark of malignancy. Recent studies showed that anoikis-resistance exacerbated malignancy of HCC cells and contributed to HCC progression,^[9–12]^ indicating that discovery of differentially expressed miRNAs in the course of anoikis-resistance may be used as potential select strategy for HCC diagnosis.

In this study, the sera from HCC patients and the supernatants from anoikis-resistant HCC cellular models were both used for screening candidate miRNAs. Next, candidate miRNAs were measured in sera samples of HCC patients and healthy controls by quantitative real-time reverse transcription-polymerase chain reaction (qRT-PCR), and their potential use as markers for the diagnosis of HCC were assessed and compared with AFP. Thus, we established a 3-miRNA signature (miR-92-3p, miR-107, and miR-3126-5p) through logistic regression analysis, and demonstrated its superiority over AFP, especially for the early diagnosis of HCC.

## 2 Methods

### 2.1 Patients and study design

Blood samples from 115 HCC patients and 40 healthy controls were collected from Provincial Hospital Affiliated to Shandong University from March 2012 to November 2013. Characteristics of these participants were presented in Table 1. All of the recruited liver cancer patients have been pathologically diagnosed as HCC (Supplementary Figure 1). There was no significant difference in the distribution of age and sex between the HCC patients and healthy controls. This study was approved by the Ethics Committee and Board of Director of the Shandong University, and written informed consent was obtained from all of these participants. Blood samples were obtained by venous puncture, allowed to be clotted for 30 minutes and centrifuged at 2000 rpm for 10 minutes to get the sera sample. The sera were then collected and allocated into new tubes and stored at −80 °C until use. Collection and manipulation of the samples were in accordance with relevant approved guidelines.

**Table 1:**
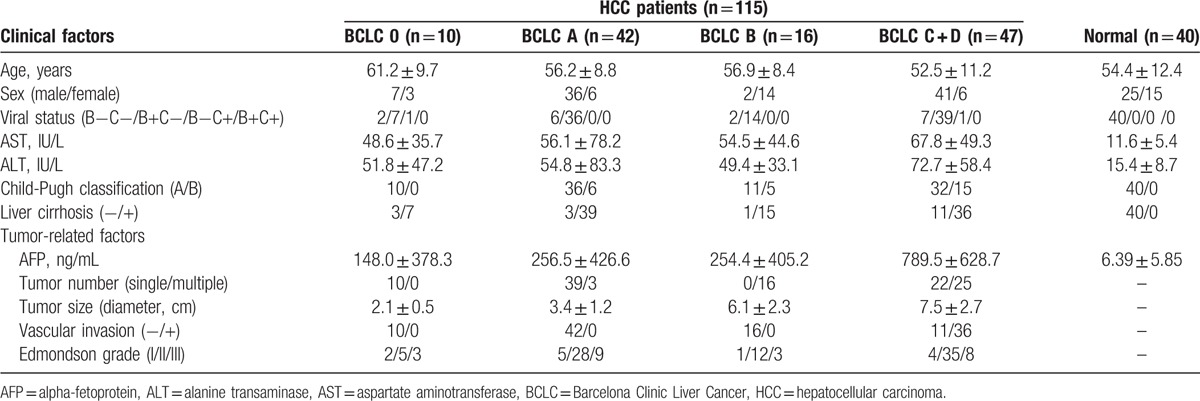
Chinicopathological characteristics of patients with hepatocellular carcinoma and healthy volunteers.

Supplementary Figure 2, shows the schematic flow chart for the candidate miRNAs of the study. We developed a strategy of performing an initial genome-wide miRNA screening by microarray analysis in both the anoikis-resistant cellular model and the sera samples from clinical specimen, and then validated the candidate genes by qRT-PCR.

#### 2.1.1 Screening out of the candidate miRNAs in HCC by microarray

First, the miRNA expression profile from the culture media of 3 anoikis-resistant HCC cell lines (BEL7402, SMMC7721, and HepG2) was compared with those of their attached counterparts. From the differentially expressed miRNAs, 13 miRNAs (including 10 upregulated and 3 downregulated miRNAs) with *P* value < 0.05 and fold expression change >1.5 were identified between these 2 statuses. Second, these 13 differentially expressed miRNAs were further defined in the clinical sera samples. Only those miRNAs verified in the clinical sera samples were served as the candidate miRNAs for further analysis. Finally, 6 candidate miRNAs (including 4 upregulated miRNAs: miR-92a-3p, miR-17-5p, miR-16-2-3p, and miR-107 and 2 down-regulated miRNA: miR-1246 and miR-3126-5p) discovered via microarrays were selected for testing by qRT-PCR.

#### 2.1.2 Confirmation of candidate miRNAs and construction of diagnostic miRNA panel

The 6 candidate miRNAs discovered via microarray were confirmed by qRT-PCR in a training cohort of sear samples from 48 participants. Five miRNAs that were differentially expressed between HCC and control groups were further tested in an extensive cohort of patients (the validation cohort), including 115 HCC patients and 40 healthy controls. These 155 participants were used to construct the diagnostic miRNAs panel based on logistic regression model for the differentiation between the HCC group and the control group.

#### 2.1.3 Evaluation of the miRNAs’ diagnostic efficacy in HCC of different stages

Using receiver-operating characteristic curve (ROC), we evaluated the diagnostic performance of the candidate miRNAs and the miRNAs panel in both the early stage (Barcelona Clinic Liver Cancer [BCLC] 0–A) and late stage (BCLC B, C, and D) HCC patients for their diagnostic capability.

### 2.2 Preparation of cell culture supernatants

HepG2, BEL7402, and SMMC7721 HCC cells were maintained in RPMI-1640 medium at 37 °C with 5% CO_2_. HCC cells were plated in poly-2-hydroxyethyl methacrylate (poly-HEMA)-coated or uncoated plates as detached and attached cells as described before.^[9,10]^ Briefly, poly-HEMA was dissolved in 95% ethanol at 36 mg/mL and added to 6 well plates to cover the surface of the plates. After casting away the ethanol, plates were rinsed with PBS for 3 times before the experiments. Cells were seeded in the plates with or without poly-HEMA as detached or attached experimental groups, respectively. We collected the supernatants from these anoikis-resistant cells and stored at −80 °C for further analysis.

### 2.3 MiRNAs microarray analysis

The differentially expressed miRNAs from the supernatants of anoikis-resistant cellular model as well as the sera from the clinical hepatocarcinoma patients were analyzed to select candidate genes for further verification in a large cohort. In total, 15 groups were included for the selection of typical malignancy derived miRNAs, including supernatants from 3 anoikis-resistant cell lines and their attached counterparts, sera samples from 3 metastatic HCC patients, 3 primary HCC patients, and 3 healthy controls. Total RNA was isolated, purified, and labeled with a miRCURY Hy3/Hy5 labeling kit (Exiqon, Denmark), and were then mixed pairwise and hybridized to the miRCURY LNA array (Exiqon, Denmark) according to its manual. The clustering analysis was done using the hierarchical method, the average linkage, and the Euclidean distance metrics.

### 2.4 MiRNAs isolation and qRT-PCR

MiRNAs were isolated from 0.4 mL of serum using miRNA isolation kit (Bioteke, Beijing, China) according to the manufacturer's protocol. Purity and concentration of RNA were determined by a dual beam UV spectrophotometer (Eppendorf AG, Hamburg, Germany). For testing of candidate miRNAs, qRT-PCR was performed using All-in-OneTM miRNA qRT-PCR Detection Kit (GeneCopoeia, Rockville, MD) according to the manufacturer's instructions. The PCR conditions were as follows: predenaturing at 95 °C for 10 minutes, followed by 40 cycles of denaturing at 95 °C for 10 seconds, annealing at 58 °C for 20 seconds, and extension at 72 °C for 10 seconds. All assays were carried out in triplicate and the expression of U6 was used as a stable endogenous control for internal normalization. The primers for realtime PCR included a specific upstream primer and a nonspecific downstream adapter primer. Primers of these detected miRNAs are listed in Supplementary Table 1.

### 2.5 Statistical analysis

Data were presented as mean ± SD. For the data obtained by qRT-PCR, the Mann–Whitney *U* unpaired test was performed to compare the difference of miRNA expression levels between HCC and control. Unsupervised hierarchical clustering analysis was performed by Cluster 3.0 software (version 3.0.111.0, Stanford University, Stanford, CA). Binary stepwise logistic regression analysis was used to determine the relationship between miRNAs and the incidence of HCC in order to build up the appropriate logistic model. The predicted probability of being diagnosed with HCC was used as a surrogate diagnostic marker to construct ROC. Area under ROC curve (AUC) was used as an accuracy index for evaluating the diagnostic efficacy of the selected miRNA. Five-fold cross-validation was used to evaluate the diagnostic performance of the selected miRNA.^[13,14]^ A 2-sided *P* < 0.05 was considered to be statistically significant.

## 3 Results

### 3.1 Candidate miRNAs screening and testing

A total of 175, 91, and 125 miRNAs were identified to be upregulated in the culture media of anoikis-resistant BEL7402, SMMC7721, and HepG2 cell lines compared with their attached counterparts, respectively. Among them, expression levels of 5 miRNAs (miR-92a-3p, miR-17-5p, miR-16-2-3p, miR-16-5p, and miR-486-5p) were simultaneously upregulated in all of the 3 HCC cell lines. Additionally, another 5 miRNAs (miR-107, miR-30e-5p, miR-140-3p, miR-7-5p, and miR-99a-5p) were significantly upregulated in the detached cells as revealed by statistical analysis (*P* < 0.05). Moreover, 253 downregulated miRNAs were confirmed in these 3 tested cell lines. Among them, 3 miRNAs (miR-20b-3p, miR-1246, and miR-3126-5p) were significantly downregulated in the anoikis-resistant cells as revealed by statistical analysis (*P* < 0.05). Next, a comparison of 3 HCC patients and controls’ sera samples identified 56 and 34 miRNAs that were significantly upregulated and downregulated, respectively. Consequently, 4 upregulated miRNAs (miR-92a-3p, miR-17-5p, miR-16-2-3p, and miR-107) and 2 downregulated miRNAs (miR-1246 and miR-3126-5p) were verified in clinical sera samples and subjected to further qRT-PCR confirmation.

### 3.2 Potential diagnostic biomarkers of 4 candidate miRNAs

**Figure 1:**
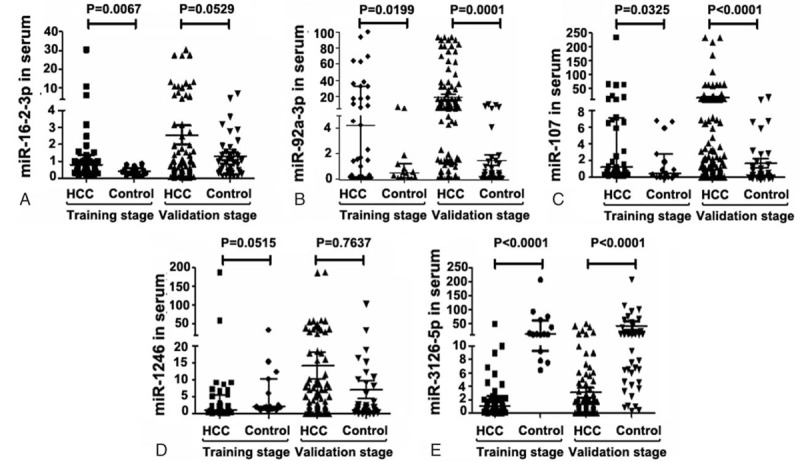
Expression of the 5 candidate miRNAs in sera from HCC patients and healthy control in the training stage and validation stage. Realtime PCR analysis was done to detect the expression level of miR-16-2-3p (A), miR-92a-3p (B), miR-107 (C), miR-3126-5p (D), and miR-3126-5p (E) in the sera samples from HCC patients and healthy control in the training stage and validation stage. HCC = hepatocellular carcinoma, miRNA = microRNA, PCR = polymerase chain reaction.

As shown in Fig. 1, 5 miRNAs (miR-16-2-3p, miR-92a-3p, miR-107, miR-1246, and miR-3126-5p) showed a significant expression difference between HCC patients and healthy control in the training cohort. Then, these 5 candidate miRNAs were further confirmed in the validation cohort. Except for miR-1246, the differential expression patterns of other 4 candidate miRNAs were consistent with the result from the training cohort, suggesting that these 4 miRNAs (miR-16-2-3p, miR-92a-3p, miR-107, and miR-3126-5p) in sera might serve as potential diagnostic biomarkers for HCC.

### 3.3 Diagnostic value of the candidate miRNAs for HCC

**Figure 2:**
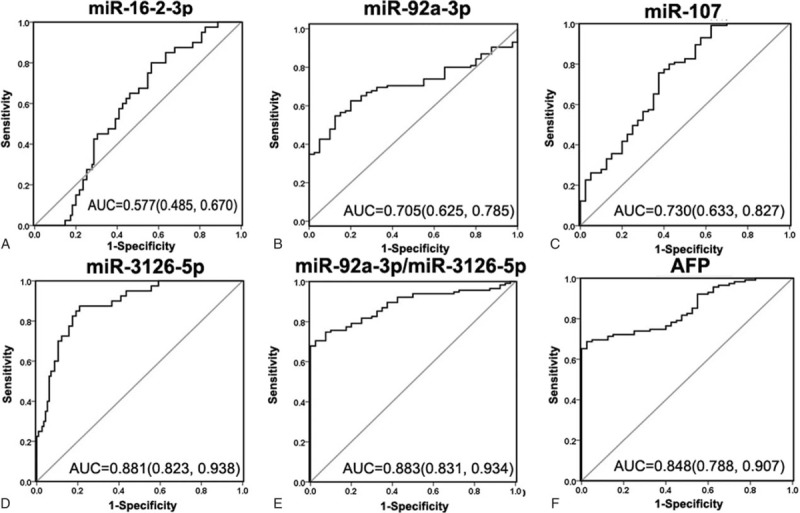
ROC curves analysis for the HCC diagnostic capability of the serum candidate miRNAs. AUC estimation for the serum concentration miR-16-2-3p (A), miR-92a-3p (B), miR-107 (C), miR-3126-5p (D), the ratio of miR-92a-3p/miR-3126-5p (E), and AFP (F) in discriminating HCC patients (n = 115) from healthy control (n = 40). AFP = alpha-fetoprotein, AUC = area under ROC curve, HCC = hepatocellular carcinoma, miRNA = microRNA, ROC = receiver-operating characteristic curve.

Using ROC analysis, we found that miR-3126-5p showed the best diagnostic efficacy with the highest AUC value (AUC = 0.881), followed by miR-107(AUC = 0.730) and miR-92a-3p (AUC = 0.705, Fig. 2B–D). However, miR-16-2-3p (AUC = 0.577, Fig. 2A) was unable to distinguish HCC patients from healthy controls and was not considered for further study. Additionally, we also evaluated the diagnostic efficacy of the ratio of miR-92a-3p/miR-3126-5p. The AUC score of this model was slightly higher than that of AFP (0.883, 95% confidence interval [CI] = 0.831–0.934 vs 0.848, 95%CI = 0.788–0.907, Fig. 2E and F). Altogether, these AUC analysis data showed that diagnostic efficacy of miR-3126-5p and the ratio of miR-92a-3p/miR-3126-5p were superior to that of AFP.

### 3.4 Diagnostic performance of the candidate miRNAs in different stages of HCC patients

**Figure 3:**
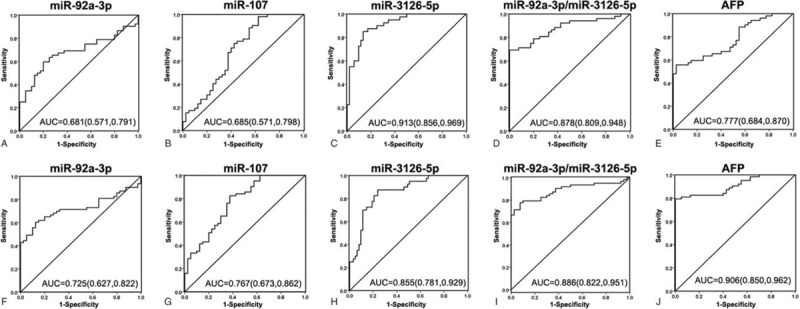
ROC analysis of the candidate miRNAs for the diagnostic ability of HCC in different stages of HCC. AUC of the candidate miRNAs, including miR-92a-3p (A), miR-107 (B), miR-3126-5p (C), the ratio of miR-92a-3p/miR-3126-5p (D), and AFP (E) were analyzed for discriminating the early stage HCC patients (BCLC 0–A) from the healthy control. AUC of miR-92a-3p (F), miR-107 (G), miR-3126-5p (H), the ratio of miR-92a-3p/miR-3126-5p (I), and AFP (J) were analyzed for discriminating the later stage HCC patients (BCLC B, C, and D) from the healthy control. AFP = alpha-fetoprotein, AUC = area under ROC curve, BCLC = Barcelona Clinic Liver Cancer, HCC = hepatocellular carcinoma, miRNA = microRNA, ROC = receiver-operating characteristic curve.

In the early stage (BCLC 0-A), the diagnostic efficacy of miR-3126-5p (AUC = 0.913, 95%CI = 0.856–0.969) was markedly higher than AFP (AUC = 0.777, 95%CI = 0.684–0.870) and other miRNAs candidates (Fig. 3A–E), which indicated that miR-3126-5p had the superiority of discriminating early stage patients from healthy control. In the late stage (BCLC B, C, and D), the corresponding AUC for miR-92a-3p, miR-107, miR-3126-5p and the ratio of miR-92a-3p/miR-3126-5p were 0.725, 0.767, 0.855, and 0.886, respectively (Fig. 3F–I). Although AFP showed the best diagnostic utility with the highest AUC value in the late stage patients (0.906, 95%CI = 0.850–0.962, Fig. 3J), diagnostic value of miR-3126-5p was always effective for all stages of HCC patients (AUC = 0.913 for early stage, and AUC = 0.855 for late stage). These results indicated that miR-3126-5p was a distinctive marker for the diagnosis of HCC, especially for the diagnosis of early stage patients.

### 3.5 Establishing the predictive 3-miRNA signature and evaluating its diagnostic efficacy

As Table 2 shows, in the univariate logistic regression model, the levels of miR-92a-3p and miR-3126-5p were significantly different between HCC patients and healthy controls (both *P* < 0.05). The miR-107 was also included in further verification, although it showed as an edge significance (*P* = 0.06813).

**Table 2:**
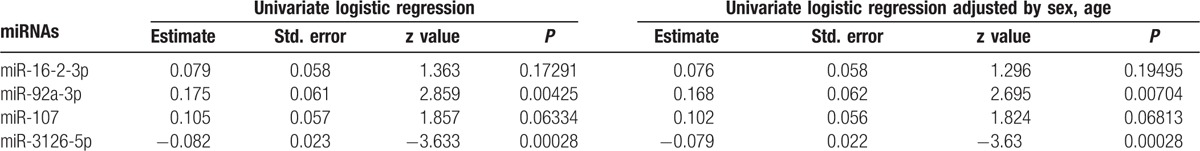
Univariate logistic regression analysis of the candidate microRNAs.

**Figure 4:**
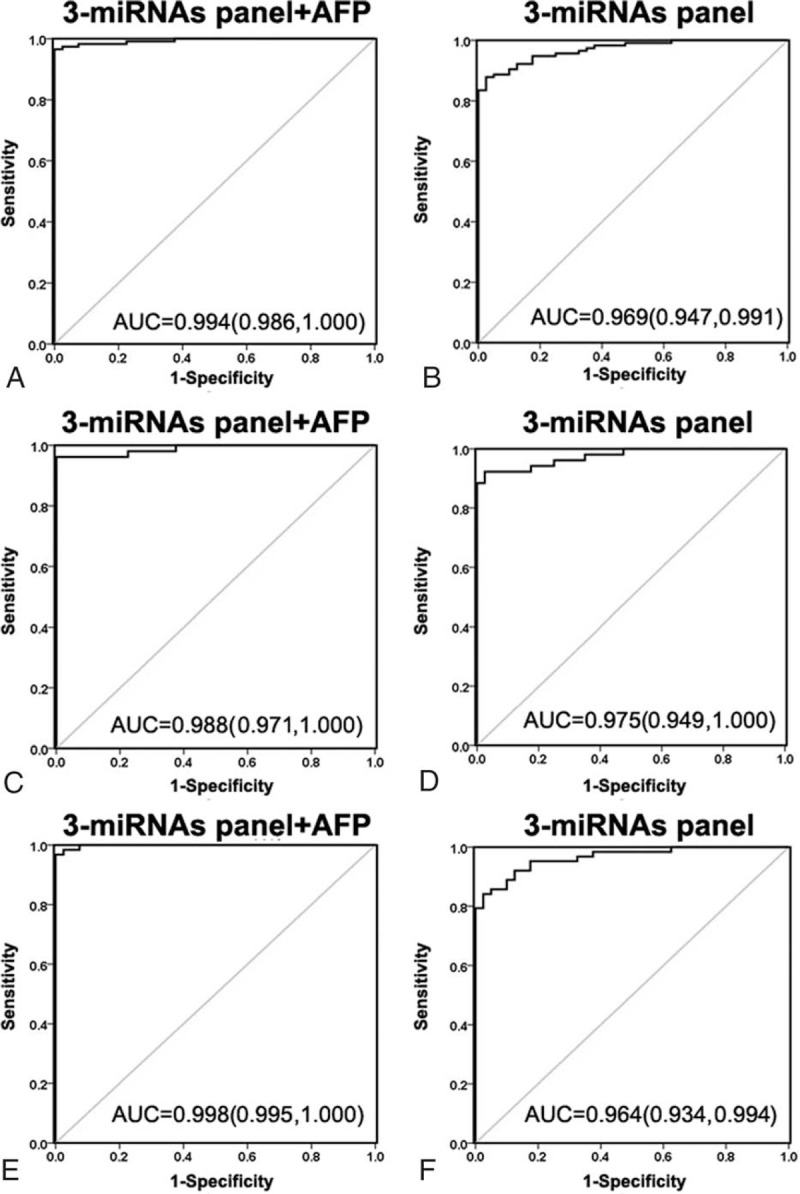
ROC analysis of logit model with 3-miRNAs panel, the combination of 3-miRNAs panel, and AFP at the different stages of HCC. (A) AUC estimation of the logit(p) value for the combination of 3-miRNA panel and AFP in discriminating the HCC patients (BCLC 0–D) from the healthy control were analyzed. (B) AUC estimation of the logit(p) value for the 3-miRNAs panel in discriminating the HCC patients (BCLC 0–D) from the healthy control were analyzed. (C) AUC estimation of the logit(p) value for the combination of 3-miRNAs panel and AFP in discriminating the early stage HCC patients (BCLC 0–A) from the healthy control were analyzed. (D) AUC estimation of the logit(p) value for 3-miRNAs panel in discriminating the early stage HCC patients (BCLC 0–A) from the healthy control were analyzed. (E) AUC were analyzed for the logit(p) value of the combination of 3-miRNAs panel and AFP in discriminating the later stage HCC patients (BCLC B, C, and D) from the healthy control. (F) AUC were analyzed for the logit(p) value of the 3-miRNA panel in discriminating the late stage HCC patients (BCLC B, C, and D) from the healthy control. AFP = alpha-fetoprotein, AUC = area under ROC curve, BCLC = Barcelona Clinic Liver Cancer, HCC = hepatocellular carcinoma, miRNA = microRNA, ROC = receiver-operating characteristic curve.

All 3 selected miRNAs turned out to be significant predictors for HCC (Table 3). The predicted probability of being diagnosed with HCC from the logit model [logit(p) = 1.468+0.293 × (miR-92-3p) + 0.433 × (miR-107) − 0.442 × (miR-3126-5p)] was used to construct the ROC curve. AUC of the 3-miRNA panel was 0.969 (95% CI = 0.947–0.991, Fig. 4B) for the diagnosis with HCC (all stages), which is higher than AFP (AUC = 0.848, 95% CI = 0.788–0.907, Fig. 2F).

**Table 3:**
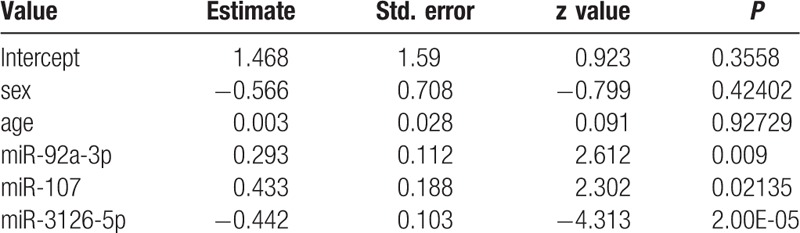
Stepwise logistic regression analysis of the selected microRNA panel.

### 3.6 Combination of 3-miRNA signature and AFP for the diagnosis of HCC at different stages

The combination of the 3-miRNA panel and AFP for discriminating HCC (all stages) from healthy control was analyzed by a stepwise logistic regression model [logit (p) = −1.545 + 0.403 × (miR-92-3p) + 0.919 × (miR-107) − 0.944 × (miR-3126-5p)+0.079 × (AFP)] (Supplementary Table 2). ROC analysis showed that the combination of AFP and the 3-miRNA panel has a significant superiority in diagnostic efficacy (AUC = 0.994, 95% CI = 0.986–1.000, Fig. 4A), compared with AFP alone (AUC = 0.816; 95% CI = 0.751–0.88, Fig. 2F) and 3-miRNA panel alone (AUC = 0.969, 95%CI = 0.947–0.991, Fig. 4B). The diagnostic value of the combination of the 3-miRNA panel and AFP was also verified in a 5-fold cross-validation (Supplementary Table 3).

The diagnostic performance of the combination (AFP and 3-miRNA panel) in different BCLC stages was further evaluated. ROC analysis showed that the combination (AFP and 3-miRNA signature) has a rather high AUC value in the early stage patients (BCLC 0 + A, 0.988, 95% CI = 0.971–1.000, Fig. 4C and D), as well as in the late stage patients (BCLC B + C + D, 0.998, 95% CI = 0.995–1.000, Fig. 4E and F). These data demonstrated that this 3-miRNA signature played a significant complementary role to AFP for the diagnosis of HCC. Thus, the combination (AFP and 3-miRNA signature) has a good diagnostic efficacy independent of disease stages and could effectively diagnose HCC patients, which made it an optimal tool for early detection of HCC.

Cases with AFP level higher than 17.4 ng/mL or probabilities by the logistic regression model higher than 0.763 were judged as a positive diagnosis for HCC. The specificity, sensitivity, accuracy, positive likelihood ratio (LR+), negative likelihood ratio (LR−), and Youden index of these predictors for diagnosis with HCC were listed in Supplementary Table 4. All the diagnostic accuracies of the combination were significantly improved compared with both AFP alone and the 3-miRNA panel because of the obvious increase in both the sensitivity and the specificity. For the full combination, especially in the early stage (BCLC 0 + A) HCC patients, the diagnostic Youden index of the combination was >96.5% with LR+ (all >26) and LR− (all = 0), which was much better than the diagnostic efficacy of both the AFP alone and the 3-miRNA panel.

### 3.7 Performance of the combination of 3-miRNA signature and AFP for the diagnosis of HCC in patients with low AFP level

**Figure 5:**
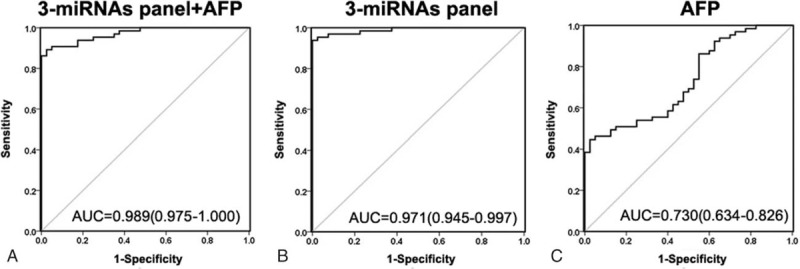
ROC analysis of the logit model for the combination of AFP and 3-miRNA panel, 3-miRNA panel alone, and AFP alone in the HCC patients with low AFP level (AFP < 200 ng/mL). (A) AUC estimation of the logit(p) value for the combination of 3 miRNAs panel and AFP in discriminating the HCC patients with low AFP level from the healthy control were analyzed. (B) AUC were analyzed for the logit(p) value for the 3 miRNAs panel in discriminating the HCC patients with low AFP level from the healthy control. (C) AUC were analyzed for the logit(p) value for AFP in discriminating the HCC patients of low AFP level from the healthy control. AFP = alpha-fetoprotein, AUC = area under ROC curve, HCC = hepatocellular carcinoma, miRNA = microRNA, ROC = receiver-operating characteristic curve.

For those patients with low AFP level, the 3-miRNA signature (AUC = 0.971, 95% CI = 0.945–0.997, Fig. 5B) has a much better diagnostic efficacy than AFP (AUC = 0.730, 95% CI = 0.634–0.826, Fig. 5C), whereas the combination (3-miRNA signature and AFP) has the highest diagnostic efficacy (AUC = 0.989, 95% CI = 0.975–1.000 Fig. 5A) by a slight superiority over 3-miRNA signature alone. These results indicated that in those HCC patients with difficulty for diagnosis because of low AFP level, the 3-miRNA signature is exponentially superior to AFP for the diagnosis of HCC.

## 4 Discussion

Tumorigenesis is a systematic disease and needs to be judged from an integrated point of view. It has been recognized recently that miRNA levels in the serum are exceptionally stable independent of age and sex of the detected subject, thus they hold great potential as disease markers in the clinical setting.^[8]^ Unlike AFP with low sensitivity at early HCC stages, specific change of circulating miRNAs may appear early in cancer as a mechanism for functional regulation and early diagnosis. Compared with traditional diagnostic strategies, detection of miRNAs in sera or plasma is thought to be a novel noninvasive diagnosis strategy with great potential for clinical application.^[15]^ In this study, we provided a unique approach to identify a particular serum miRNA profile for early diagnosis of HCC. Initially, we screened the candidate HCC-specific miRNAs by Exiqon miRNA microarray through the combination of clinical sera samples and the anoikis-resistant HCC cellular model for the first time, which enabled us to have better chance to identify candidate miRNAs which possess the most malignant characteristics of malignancy. Then, we performed qRT-PCR validation using a large and independent cohort to verify the expression difference of these candidate miRNAs. Logistic regression analysis was further performed, thus we obtained a 3-miRNA panel (miR-92-3p, miR-107, and miR-3126-5p) which shows more encouraging diagnostic utility in early detection of HCC and in patients with low AFP level. In this way, we identified a unique expression profile of sera miRNAs for the diagnosis of HCC.

In the conventional screening method, the candidate miRNAs were screened through searching for the differentially expressed miRNAs in a wide spectrum of patients and controls.^[16,17]^ It was difficult to avoid the individual difference and background variation, thus the reproducibility and repeatability of the acquired data were relatively not guaranteed. Acquisition of anoikis-resistance is recognized as a hallmark of malignancy,^[18,19]^ and anoikis-resistant cells presented the most distinguishable characteristics for malignancy. Thus, this approach based on the anoikis-resistant cellular model of HCC is unique for several distinguished features, including best representation of malignancy, good reproducibility, innocent background, low variation, and cheap detective cost.

Our study demonstrated that serum miR-3126-5p was a valuable biochemical marker for HCC, especially in early diagnosis. Although the exact reason for this observation was not clear, it indicated that miR-3126-5p played an important role in tumorigenesis of early HCC. However, the process of tumorigenesis usually involves multiple pathological changes, which suggests that a single biomarker may not be sufficient to reflect the entire process. Thus, a panel of biomarkers is usually needed to enhance the diagnosis efficiency for early stage HCC. In this study, we demonstrated that the diagnostic capability of the 3-miRNA panel (miR-92a-3p, miR-107, and miR-3126-5p) from the multivariate logistic regression model had better diagnostic efficacy than any single miRNA, and this panel could effectively supplement AFP for diagnosing early stage patients.

AFP is frequently used for the diagnosis of HCC, including onset and recurrence of HCC after liver transplant.^[20]^ However, approximately 20% of HCC patients with low AFP level are poorly diagnosed.^[21]^ For these patients, the 3-miRNA panel possessed significant superiority compared to AFP alone. For some indolent HCC without any image change, diagnosis for HCC is rather challengeable and AFP is the most commonly applied strategy for the diagnosis of HCC. Although some new markers, such as DCP and AFP-L3, were recognized to have better diagnostic efficacy for HCC,^[22,23]^ these new markers have not been widely validated in routine clinical practice. In those patients with low AFP level, diagnosis of HCC is rather difficult and inaccurate. In this situation, the evaluation of our 3-miRNA signature showed significantly better diagnostic performance than AFP.

Recent publication showed that some miRNAs, including miR-200 family and other miRNAs, have good diagnostic efficacy for HCC;^[24,25]^ however, our study has distinguishable characteristics compared with previous reports for the following reasons: This study is the first report to take both of the clinical specimen and anoikis-resistant cellular model in consideration for the screening of the candidate miRNA; We investigate the diagnostic efficacy of these candidate miRNA in both the early stage (BCLC 0–A) HCC patients and late stage (BCLC B–D) HCC patients; The diagnostic efficacy of these candidate miRNAs was investigated in HCC patients with low AFP level; and This study investigated the diagnostic efficacy of the combination of 3-miRNA panel and AFP, which offered more alternative strategy for the diagnosis of HCC. Because of the limited amount of specimens of hepatocellular adenomas,^[26]^ the diagnosis efficiency of 3-miRNA panel among HCC and adenomas did not evaluated in this study. Additionally, some studies have reported that miRNAs have great potential as biomarkers for the diagnosis of cholangiocarcinomas.^[27]^ Further studies may be required to evaluate the diagnosis efficiency of 3-miRNA panel in these solid hepatic lesions.

In conclusion, we have defined the distinctive serum miRNA signature for the early diagnosis of HCC. Our data demonstrated that the unique 3-miRNA signature (miR-92a-3p, miR-107, and miR-3126-5p) combined with AFP can serve as a sensitive, specific, and noninvasive biomarker for the diagnosis of HCC, especially in the patients at early stages or with low AFP level. Although further validation using a larger amount of patients or additional correlation analysis between miRNAs signatures and long-term patient outcome is required, the miRNA panel identified in our study showed great potential as noninvasive biomarkers for HCC.

## Supplementary Material

Supplemental Digital Content

## Supplementary Material

Supplemental Digital Content

## Supplementary Material

Supplemental Digital Content
