# Supplementary material for: Serum microRNA panel for early diagnosis of the onset of hepatocellular carcinoma
Source: Medicine (Baltimore). 2017 Jan 13;96(2):e5642. doi: 10.1097/MD.0000000000005642 (PMC5266158; doi:10.1097/MD.0000000000005642)
Supplement: Supplemental Digital Content [file medi-96-e5642-s001.docx]

**Supplementary Figure 1**. Flow diagram of treatment and diagnosis for 115 patients

115 patients

84 patients

31 patients

Receiving surgical resection

surgery

Pathological diagnosis for HCC

Percutaneous liver needle biopsy guided by the ultrasound (pathological diagnosis for HCC)

Radiofrequency ablation

Transcather hepatic arterial chemoembolization (TACE)

9 patients

(BCLC 0+A)

22 patients

(BCLC B+C+D)

Conservative

Treatments

**Supplementary Figure 2.** Overview of the experimental design
