# Supplementary material for: Serum microRNA panel for early diagnosis of the onset of hepatocellular carcinoma
Source: Medicine (Baltimore). 2017 Jan 13;96(2):e5642. doi: 10.1097/MD.0000000000005642 (PMC5266158; doi:10.1097/MD.0000000000005642)
Supplement: Supplemental Digital Content [file medi-96-e5642-s002.docx]

**Supplementary Figure 2.** Overview of the experimental design

**Biomarker-discovery stages:** **Exiqon Sequencing using anoikis-resistance HCC cellular model and validation in serum samples.**

*Selection criteria*：miRNAs have at least 50 copies in the samples and a concentration altered 1.5-fold

**Biomarker-training stages: Individual qRT-PCR**

Selection criteria: miRNAs have mean changes more than 1.5 fold, p<0.05.

34 HCC case *vs.* 14 healthy controls

**Biomarker-validation stages: Individual qRT-PCR (including the training stages samples)**

Selection criteria: miRNAs have mean changes more than 1.5 fold, p<0.05.

115 HCC case *vs.* 40 healthy controls

**The panel of 4 miRNAs as a** **candidate biomarker for HCC diagnosis**

(Upregulated: MiR-16-2-3p; MiR-92a-3p; MiR-107

Downregulated: MiR-3126-5p)

3 miRNAs↑1 miRNAs↓

3 miRNAs↑2 miRNAs↓

5 miRNAs↑3 miRNAs↓
