# Supplementary material for: Serum microRNA panel for early diagnosis of the onset of hepatocellular carcinoma
Source: Medicine (Baltimore). 2017 Jan 13;96(2):e5642. doi: 10.1097/MD.0000000000005642 (PMC5266158; doi:10.1097/MD.0000000000005642)
Supplement: Supplemental Digital Content [file medi-96-e5642-s003.doc]

**Supplementary Table 1.** Specific upstream primers for realtime PCR assay of candidate microRNA

|  | **Specific upstream primer** |
| --- | --- |
| miR-16-2-3p | CCAATATTACTGTGCTGCTTTA |
| miR-92a-3p | TATTGCACTTGTCCCGGCCTGT |
| miR-107 | AGCAGCATTGTACAGGGCTATCA |
| miR-1246 | AATGGATTTTTGGAGCAGG |
| miR-3126-5p | TGAGGGACAGATGCCAGAAGCA |
| miR-17-5p | CAAAGTGCTTACAGTGCAGGTAG |

**Supplementary Table 2.** Stepwise logistic regression analysis of the 3-microRNA panel and AFP

| **Value** | **Estimate** | **Std. Error** | **z value** | **P value** |
| --- | --- | --- | --- | --- |
| Intercept | -1.545 | 2.78 | -0.556 | 0.57834 |
| sex | -1.194 | 1.399 | -0.853 | 0.39351 |
| age | 0.038 | 0.05 | 0.757 | 0.44876 |
| miR-92a-3p | 0.403 | 0.223 | 1.808 | 0.07067 |
| miR-107 | 0.919 | 0.542 | 1.695 | 0.09007 |
| miR-3126 | -0.944 | 0.285 | -3.31 | 0.00093 |
| AFP | 0.079 | 0.042 | 1.874 | 0.06096 |

**Supplementary Table 3.** ROC analysis of the AUC based on five-fold cross validation

| MicroRNA | threshold | AUC(95%CI) | Sensitivity(95%CI) | Specificity(95%CI) |
| --- | --- | --- | --- | --- |
| AFP | 0.696 | 0.816 (0.751, 0.88) | 0.687 (0.6, 0.765) | 0.975 (0.925, 1) |
|  |  |  |  |  |
| 3-MiRNA panel | 0.818 | 0.962(0.935, 0.988) | 0.878 (0.817, 0.93) | 0.975 (0.925, 1) |
|  |  |  |  |  |
| Combination of 3-MiRNA panel and AFP | 0.743 | 0.99 (0.98, 1) | 0.957 (0.913, 0.991) | 0.975 (0.925, 1) |

**Supplementary Table 4. Differentiating power of AFP, microRNA, and the combination of AFP and microRNA**

|  | | **Sensitivity (95%CI)** | **Specificity (95%CI)** | **Youden index** | **LR+** | **LR-** |
| --- | --- | --- | --- | --- | --- | --- |
| HCC *vs.* Normal | AFP alone | 97.5%(86.8-99.9) | 68.7%(59.4-77.0) | 66.2% | 3.11 | 0.036 |
|  | 3-miRNA panel | 97.5%(86.8-99.9) | 87.8%(80.4-93.2) | 85.3% | 8.01 | 0.028 |
|  | Combination of 3-miRNA panel and AFP | 100%(91.2-100.0) | 96.5%(91.3-99.0) | 96.5% | 28.70 | 0.00 |
| HCC  BCLC (0+A)  *vs.* Normal | AFP alone | 97.5%(86.8-99.9) | 55.8%(41.3-69.5) | 53.3% | 2.20 | 0.045 |
|  | 3-miRNA panel | 97.5%(86.8-99.9) | 92.3%(81.5-97.9) | 89.8% | 12.60 | 0.027 |
|  | Combination of 3-miRNA panel and AFP | 100%(91.2-100.0) | 96.2%(86.8-99.5) | 96.2% | 26.00 | 0.00 |
| HCC  BCLC (B+C+D)  *vs.* Normal | AFP alone | 100%(91.2-100.0) | 79.4%(67.3-88.5) | 79.4% | 4.85 | 0.00 |
|  | 3-miRNA panel | 97.5%(86.8-99.9) | 84.1%(72.7-92.1) | 81.6% | 6.14 | 0.03 |
|  | Combination of 3-miRNA panel and AFP | 100%(91.2-100.0) | 96.8%(89.0-99.6) | 96.8% | 31.5 | 0.00 |
